# Diverse preferences, different solutions: Exploring remote monitoring preferences in Parkinson's disease through a discrete choice experiment

**DOI:** 10.1177/1877718X251327752

**Published:** 2025-03-24

**Authors:** Carlos Antonio Godoy Junior, Laura Mäkitie, Eleonora Fiorenzato, Maija Koivu, Joonas Niskala, Angelo Antonini, Lytske Jantien Bakker, Luis Pilli, Carin Uyl-de Groot, William Ken Redekop, Welmoed Kirsten van Deen

**Affiliations:** 1 Erasmus School of Health Policy and Management, Erasmus University Rotterdam, Rotterdam, Netherlands; 2 Erasmus Choice Modeling Centre, Erasmus University Rotterdam, Rotterdam, Netherlands; 3 Department of Neurology and Department of Clinical Neurosciences (Neurology), University Hospital and University of Helsinki, Helsinki, Finland; 4 Department of Neuroscience, University of Padova, Padova, Italy; 5 Padua Neuroscience Center (PNC), University of Padua, Padua, Italy; 6 Parkinson and Movement Disorders Unit, Study Center for Neurodegeneration (CESNE), Department of Neuroscience, University of Padua, Padua, Italy

**Keywords:** Parkinson's disease, movement disorders, remote sensing technology, remote patient monitoring, telemedicine, patient preference

## Abstract

**Background:**

Remote monitoring solutions (RMS) have the potential to improve Parkinson's disease (PD) management by enabling continuous symptom tracking and personalized care. Understanding patient preferences for RMS features is essential for successful implementation.

**Objective:**

This study aimed to investigate the preferences of people with Parkinson's disease (PwP) for RMS features and identify preference heterogeneity across distinct patient subgroups.

**Methods:**

From November 2023 to February 2024, a discrete choice experiment (DCE) was conducted among PwP in Finland and Italy to elicit preferences for RMS attributes, including monitoring frequency, time spent filling questionnaires, home video recordings, and clinical benefits (delay in advanced symptom onset). Latent class analysis (LCA) was used to identify subgroups with distinct preference patterns, and adoption probabilities under varying RMS scenarios were estimated.

**Results:**

A total of 411 PwP participated, revealing significant heterogeneity in RMS preferences. While clinical benefits, particularly delaying advanced symptom onset, were the most valued attribute overall, preferences diverged across subgroups. Some participants strongly preferred home video recordings, whereas others expressed aversion to this feature. A smaller subgroup exhibited reluctance toward RMS adoption, regardless of its benefits.

**Conclusions:**

PwP generally view RMS favorably, but preferences for specific features vary substantially across subgroups. Clinical benefits are a key driver of adoption, while home video recordings elicit both strong preference and aversion, highlighting the impracticality of a one-size-fits-all approach. Tailoring RMS to diverse patient needs, addressing concerns, and enhancing usability through customization are essential for successful implementation and widespread acceptance in PD management.

## Plain language summary

New technology, like smartwatches and home cameras, can help doctors track Parkinson's disease symptoms at home and adjust treatment when needed. But before using these tools, it's important to know what persons with Parkinson's disease feel comfortable with—for example, would they wear a smartwatch all day, agree to home recordings, or be okay with answering doctor's surveys? To find out, 411 persons with Parkinson's disease in Finland and Italy took part in a survey. They chose between different options, like how often monitoring happens, how much time they spend on questionnaires, whether they’re okay with home video recordings, and what health benefits they expect. The results showed that most participants were open to using digital monitoring at home if it could improve their health. However, opinions on home video recordings were mixed—those with more symptoms or greater difficulties at home were more accepting, while others preferred not to be recorded. A small group did not want to use remote monitoring at all. These findings suggest that while many persons with Parkinson's disease support digital symptom tracking, the technology should be flexible and easy to use. Offering customizable options will help ensure that persons with Parkinson's disease can benefit from better care while feeling comfortable with the technology.

## Introduction

Parkinson's disease (PD) is a long-term, progressively debilitating disorder affecting over 8.5 million people globally as of 2019,^1–3^ with numbers projected to exceed 12 million by 2040 worldwide.^4^ PD manifests through a range of motor symptoms (e.g., tremors, stiffness) and non-motor symptoms (e.g., anxiety, cognitive decline decline).^1^ Current treatments focus on managing symptoms as no disease-modifying therapies exist.^5^

Personalizing treatments for persons with PD (PwP) remains challenging due to the disease's variable progression and the lack of definitive biomarkers to identify disease progression.^5,6^ Treatment decisions rely on clinical assessments, symptom questionnaires, and rating scales,^7^ which are often subjective and dependent on PwP recall, potentially leading to delayed symptom identification and suboptimal care.^8^

Recently, interest has grown in using technology for more objective PD symptom evaluation.^9,10^ Remote monitoring solutions (RMS), including wearable devices like smartwatches and non-wearable systems such as medication dispensers and home surveillance, have been utilized in clinical research.^11–14^ These technologies allow continuous symptom monitoring, offering a more objective basis for treatment adjustments and potentially enabling proactive, artificial intelligence (AI)-based care strategies capable of efficiently processing large volumes of data to aggregate, detect, track, and predict symptom progression.^15^

Despite RMS's potential to improve clinical assessments, their adoption in routine care faces obstacles.^16^ Many PwP perceive these devices as intrusive or uncomfortable for everyday use and are uncertain about their benefits.^17–19^ Thus, while promising, RMS's real-world application requires addressing these usability concerns. Currently, quantitative evidence also remains limited. This study seeks to provide measurable insights into RMS preferences in PD, identifying the features most valued by patients. By incorporating patient-centered data, the findings aim to inform the design and implementation of RMS to better align with user needs and expectations.

## Methods

To evaluate PwP preferences for RMS in PD, this study employed a discrete choice experiment (DCE). The DCE, a well-established cross-sectional survey method introduced to healthcare research in the early 1990s,^20^ was conducted at Helsinki University Hospital (HUS) in Finland and the University of Padua (UNIPD) in Italy, both partners in the EU-funded AICCELERATE project, which focuses on advancing AI-driven healthcare technologies.^21^

PwP were recruited from November 2023 to February 2024. Recruitment efforts utilized multiple channels, including in-person invitations during clinical appointments at participating hospitals and digital invitations disseminated through the Finnish Movement Disorders Association, Brain Hub, Parkinson Italia, and local patient associations.

Before participation, all individuals received information about the study and its objectives. Informed consent was obtained from all participants. Participation was voluntary and uncompensated. The study was conducted in accordance with the ethical guidelines of the Declaration of Helsinki and the Medical Research Involving Human Subjects Act (WMO).^22^ Ethical approvals were obtained from the local ethics committees at HUS (HUS/2349/2021), UNIPD (2020-III/13.41.7), and Erasmus University Rotterdam (ETH2122-0569).

### Methodology of DCE

DCEs quantitatively evaluate preferences by presenting participants with hypothetical scenarios (choice questions) that include varying attributes of interventions or services. Participants are asked to select their preferred options in each scenario, allowing for the analysis of trade-offs between attributes.^23,24^ The design, execution, and analysis of the DCE were conducted in accordance with International Society for Pharmacoeconomics and Outcomes Research (ISPOR) good research practices for conjoint analysis established protocols.^25^

### Attributes and levels

The selection of RMS attributes began with a literature review and a qualitative study involving 27 PwP and 6 neurologists from Finland and Italy.^26^ After discussion within the research group (CG, LM, EF, WD), five key attributes were selected: monitoring frequency, time spent filling online questionnaires, home video recording, delay in onset of advanced symptoms, and out-of-pocket costs. Attribute levels were selected based on literature, expert opinion, and clinical practice to ensure a realistic range.

### Experimental design, pre-testing, and piloting

The experimental design was created using NGene 1.4 software (ChoiceMetrics, 2011) to generate a D-efficient design, a statistical approach that reduces required sample size and minimizes the number of choice questions per participant.^27^ The final design comprised 12 distinct choice questions to be completed by each participant, each featuring two RMS options and an opt-out choice if neither option was acceptable.^28^

Pre-testing of the DCE draft was conducted in each country using a convenience sample (*n* = 5) in “think aloud” sessions.^29^ During these sessions, researchers (EF, JN) observed participants as they verbalized their thoughts while completing the questionnaire. This process ensured the clarity and comprehensibility of the wording, attributes, levels, and choice scenarios for the target population. Based on these five participants feedback, the survey was refined to include additional details about the “delay in onset of advanced symptoms” attribute, specifically explaining how RMS could help neurologists manage symptoms and potentially delay PD progression.

Following the think-aloud sessions, the revised survey was piloted with 14 respondents. Pilot results revealed a consistent bias towards selecting the lowest-cost option, often disregarding other attributes. To address this issue, the price attribute was removed from the DCE to focus on other critical attributes. To capture willingness to pay (WTP), a separate question was included in the survey. Pilot participants were excluded from the final sample, and insights from this phase informed a revised D-efficient design.

**Figure 1.**
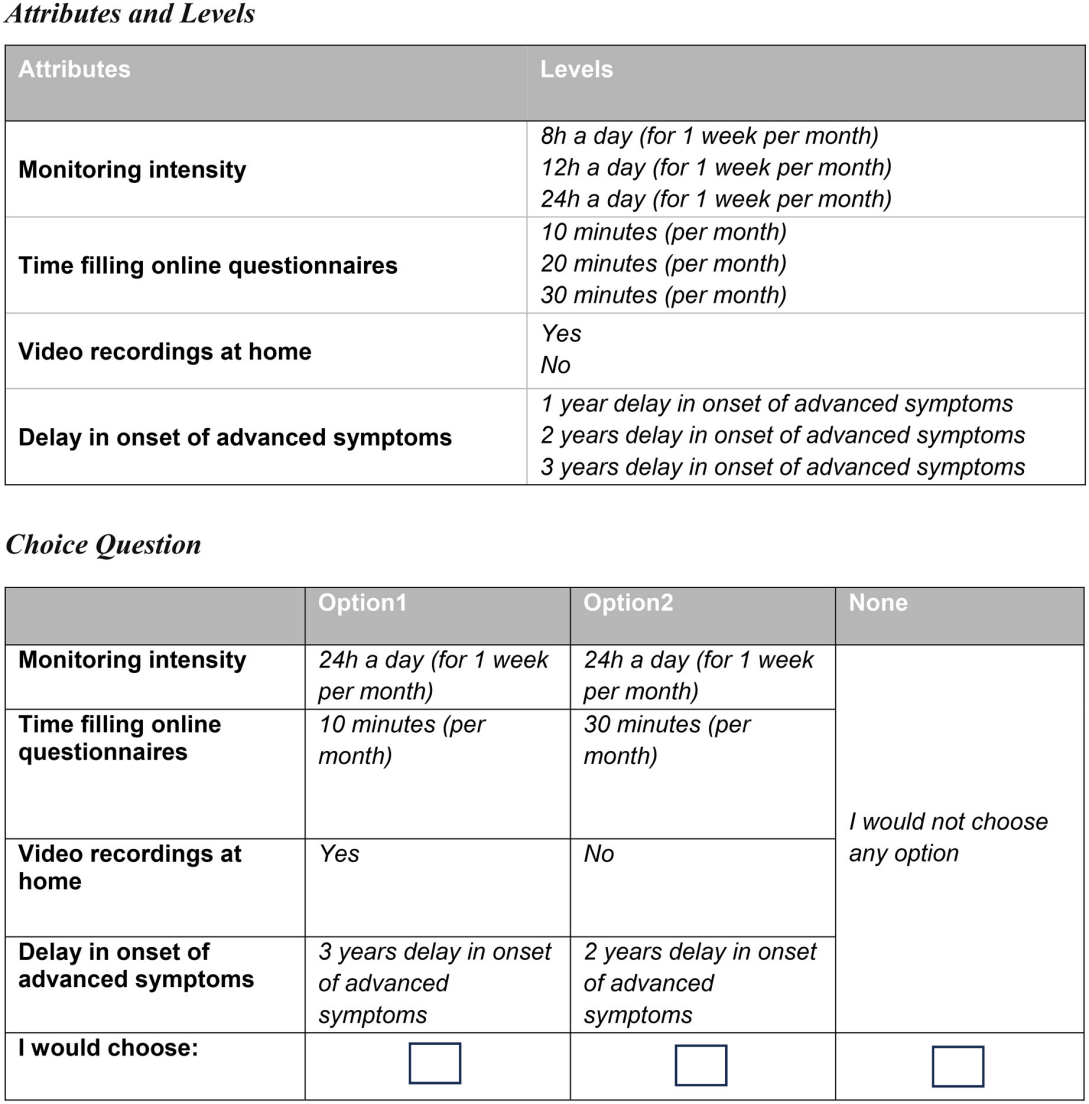
Attributes and levels included in the discrete choice experiment, and an example of one of the 12 scenarios (choice questions).

The final DCE included four attributes with 2 or 3 levels each. Figure 1 illustrates the final set of attributes and levels, along with an example of one of the 12 choice questions completed by participants.

In addition to the choice questions, the survey collected demographic and clinical data, including participants’ age, gender, education level, ethnicity, living situation, familiarity with wearable technology, data literacy, self-reported motor symptoms, number of daily levodopa dosage, disease duration, and monthly WTP for RMS usage. The Parkinson's Disease Questionnaire (PDQ-8) was also included.^30^

The questionnaire followed a structured format: introduction with study aim, informed consent, initial demographic questions, explanation of the DCE and its attributes, a warm-up question, the first six DCE questions, additional demographic questions, the remaining six DCE questions, a WTP question, quality of life questions, and a closing section. An English version of the survey is provided in Supplementary File 1.

The survey was electronically administered in Finnish and Italian using Sawtooth Software Lighthouse Studio and was designed for completion within 20–30 min. Translation was performed by a professional translator service and validated through back translation by native speakers of Finnish and Italian (LM, EF).

### Statistical analysis

Statistical analysis was conducted using R and STATA. Preferences were analyzed using multinomial logit (MNL) and latent class analysis (LCA) models in line with ISPOR guidelines.^31^ The MNL model was first applied to estimate average preferences across the entire study population,^31^ with all attributes treated as nonlinear and effect coded.^32^ Separate models were developed for the Italian and Finnish datasets to account for the multilevel data structure and assess scale heterogeneity. A heteroskedastic model was used to evaluate potential scale differences between the two samples.^33^ Analysis revealed no significant evidence of scale heterogeneity between the two countries, as detailed in Supplementary File 2. As a result, data from both countries were pooled for subsequent analyses.

LCA models were used to explore preference heterogeneity by segmenting participants into latent classes based on choice patterns.^34^ These models assume the existence of two or more unobserved classes, each associated with distinct preference structures.^31^ The optimal number of classes was determined using criteria such as the Akaike Information Criterion (AIC), Bayesian Information Criterion (BIC), Consistent Akaike Information Criterion (CAIC), and Log-Likelihood (LL),^31^ ensuring each class comprised at least 10% of the sample.^35^ Models specifying one to six classes were tested, and a four-class solution was identified as the best fit (Supplementary File 3). Following class stratification, we estimated the probability of a respondent being assigned to each class.^36^ Demographic and clinical variables were then examined for their influence on class membership (Supplementary File 4).

### Preferences

Results from the MNL and LCA models were expressed as beta coefficients, where the sign indicates whether an attribute level positively or negatively influences preferences.^31^ The magnitude of these coefficients determined attribute importance, reflecting their impact on participants’ choices.^31^ In the LCA model, the four classes were assigned descriptive nicknames to highlight their distinct priorities for RMS features, illustrating the role of these attributes in shaping preference patterns across the classes. Summing the beta coefficients under various RMS scenarios yielded class-specific utility scores. These scores were used to estimate the probability of RMS acceptance under the base scenario: monitoring frequency of 8 h daily, monthly 10-min online questionnaires, no home video recording, and a clinical benefit of delaying symptom progression by one year and to assess the marginal effects of modifying the most important attributes for each class on acceptance probabilities.^23^ Details of these calculations are provided in Supplementary File 5.

## Results

### Respondents

Of the 1124 individuals who accessed the digital questionnaire, 411 successfully completed the survey (279 in Finland and 132 in Italy). The participants were, on average, 67.9 years old. Males represented 53% of the sample and 98% identified as white (Caucasian). Of the respondents, 50% had been living with PD for five years or less, 59% were undergoing treatment with more than two daily doses of levodopa, and 50% reported experiencing mild symptoms. Furthermore, 73% of the respondents reported high digital literacy, and 33% declared prior experience with wearable technologies. The average WTP per month for the use of RMS was 49 Euros. Detailed characteristics of the study sample are presented in Table 1.

**Table 1.** Participants characteristics.

Sample characteristics	Total (*n* = 411)	Finland (*n* = 279)	Italy (*n* = 132)
Age, years [mean (SD), range]	67.9 (8.7), 26–90	68.1 (9.2), 26–90	67.4 (7.6), 40–85
Sex [*n* (%)]			
Female	193 (47%)	139 (50%)	54 (41%)
Male	218 (53%)	140 (50%)	78 (59%)
Ethnicity [*n* (%)]			
White (Caucasian)	402 (98%)	276 (99%)	128 (98%)
Number of daily levodopa dosages [*n* (%)]			
2 times/day or less	95 (23%)	65 (23%)	30 (23%)
3	102 (25%)	73 (26%)	29 (22%)
4	102 (25%)	80 (29%)	22 (17%)
5	59 (15%)	30 (11%)	29 (22%)
More than 5	50 (12%)	31 (11%)	19 (15%)
Current symptoms, self-reported [*n* (%)]			
Mild	207 (51%)	154 (55%)	53 (40%)
Moderate	161 (39%)	108 (39%)	53 (40%)
Advanced	43 (10%)	17 (6%)	26 (20%)
Duration of Parkinson's disease [*n* (%)]			
Less than 2 years	65 (16%)	40 (14%)	25 (20%)
2–5 years	139 (34%)	110 (39%)	29 (23%)
6–10 years	120 (30%)	80 (29%)	40 (31%)
More than 10 years	83 (20%)	49 (18%)	34 (26%)
Living situation [*n* (%)]			
Living in a household	337 (82%)	217 (78%)	120 (92%)
Living alone	73 (18%)	62 (22%)	11 (8%)
Level of education [*n* (%)]			
Elementary/Middle School/High School	118 (19%)	32 (11%)	86 (66%)
Professional Education, Vocational Training, or University	291 (71%)	247 (89%)	44 (34%)
PDQ8, score [mean (SD), range]	9.7 (5.9), 0–29	8.8 (5.6), 0–27	8.8 (5.6), 0–27
Digital literacy [*n* (%)]			
Low	46 (11%)	16 (6%)	30 (23%)
Medium	65 (16%)	16 (6%)	49 (37%)
High	299 (73%)	247 (88%)	52 (40%)
Experience with wearables [*n* (%)]			
Yes	137 (33%)	98 (35%)	39 (27%)
WTP (€) monthly for the use of the service [mean (SD), range]	49 (86), 0–1000	50 (99), 0–1000	46 (53), 0–300

### Overall preferences

Preferences across the entire study population revealed that two attributes significantly influenced respondents’ decision-making: the time required to complete online questionnaires and the delay in the onset of advanced symptoms. On average, PwP preferred RMS options that minimized time spent on online questionnaires and maximized delays in the onset of advanced symptoms. Conversely, respondents were generally indifferent to attributes such as monitoring frequency and home video recordings (Table 2).

**Table 2.** (Betas) coefficients estimates of multinomial logit (MNL) and latent class analysis (LCA) with four classes on respondents’ preferences for RMS features.

Model		MNL^a^		LCA^b^							
Population/Class		Overall		Class 1		Class 2		Class 3		Class 4	
Average class probability		—		45%		32%		12%		11%	
Attributes	Levels	Beta coefficient	SE	Beta coefficient	SE	Beta coefficient	SE	Beta coefficient	SE	Beta coefficient	SE
Monitoring frequency	8 h a day	Ref.		Ref.		Ref.		Ref.		Ref.	
	12 h a day	−0.02	0.05	0.05	0.28	−0.08	0.12	−0.61**	0.23	−0.96**	0.32
	24 h a day	−0.14	0.07	0.16	0.20	−0.28*	0.11	−0.60*	0.27	−1.65**	0.33
Time filling online questionnaires	10 min	Ref.		Ref.		Ref.		Ref.		Ref.	
	20 min	0.05	0.03	−0.14	0.16	0.06	0.08	−0.17	0.21	−0.52	0.30
	30 min	−0.13**	0.04	−0.17	0.14	−0.08	0.09	−0.33	0.22	−0.50	0.28
Video recordings at home	No	Ref.		Ref.		Ref.		Ref.		Ref.	
	Yes	−0.03	0.06	−0.02	0.15	0.66**	0.09	0.24	0.21	−3.11**	0.39
Delay in onset of advanced symptoms	1 year delay	Ref.		Ref.		Ref.		Ref.		Ref.	
	2 years delay	0.68**	0.07	2.94**	0.22	−0.04	0.08	0.48	0.38	0.27	0.23
	3 years delay	1.33**	0.10	5.32**	0.33	−0.11	0.09	1.00**	0.39	0.90**	0.55
Opt-out		−0.62**	0.13	−1.48**	0.35	−2.26**	0.21	1.84**	0.43	−3.07**	0.55

*Significant at the 5% level; **Significant at the 1% level.

^a^ MNL Model: Estimates the average preferences of the entire study population.

^b^ LCA Model: Identifies preference heterogeneity by segmenting respondents into classes, each representing distinct preference patterns.

### Preferences heterogeneity

Segmenting participants into four classes based on choice patterns revealed significant heterogeneity in preferences (Table 2).

**Figure 2.**
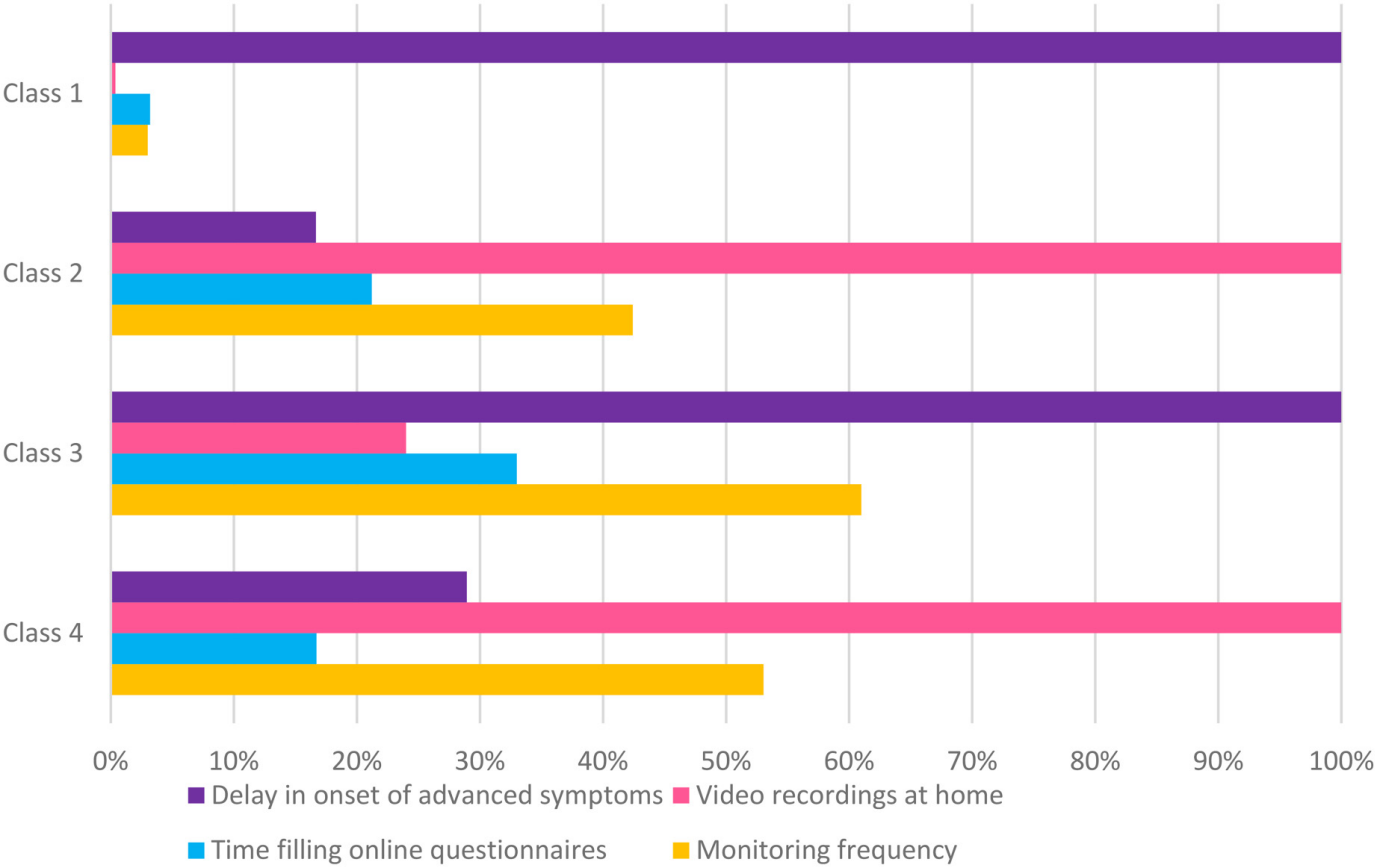
Relative importance of RMS attributes across the four classes. **Bar Length:** The length of each bar represents the relative importance of the corresponding attribute within each class. Longer bars indicate the most influential attribute in participants’ decision-making, while shorter bars reflect the importance of other attributes relative to the most significant one.

Class 1, comprising 45% of the sample and labeled “Benefit Seekers”, prioritized the clinical benefit of RMS as the most influential attribute, while showing little concern for other features (Table 2, Figure 2). Respondents in this class were characterized by younger age, fewer symptoms, lower daily levodopa dosage, shorter disease duration, higher health literacy, and greater WTP for RMS (Table 3).

**Table 3.** Classes characteristics.

Characteristic	Class 1: Benefit Seekers	Class 2: Follow Me	Class 3: Monitoring Skeptics	Class 4: Privacy-Conscious
Sample Size [*n* (%)]	185 (45%)	133 (32%)	48 (12%)	45 (11%)
Most important attribute	Delay in onset of advanced symptoms	Video recordings at home	Delay in onset of advanced symptoms	Video recordings at home
Influence of Most Important Attribute in decision making	Positive	Positive	Positive	Negative
Probability of adopting RMS				
Base case scenario^a^	81%	90%	13%	95%
Video monitoring at home scenario^b^	81%	94%	16%	49%
3-years delay in onset of advanced symptoms scenario^c^	99%	89%	30%	98%
Demographics				
Gender [*n* (%)]*				
Female	95 (51%)	54 (41%)	18 (38%)	24 (53%)
Male	90 (49%)	79 (59%)	30 (62%)	21 (47%)
Age, years [mean (SD)]*	66 (8.5)	69 (8.7)	71 (8.9)	67 (8.0)
Current symptoms, self-reported [*n* (%)]*				
Mild	105 (57%)	56 (42%)	25 (52%)	21 (47%)
Moderate	63 (34%)	61 (46%)	17 (35%)	20 (44%)
Advanced	17(9%)	16 (12%)	6 (13%)	4 (9%)
Number of daily levodopa dosages [*n* (%)]*				
2 times/day or less	53 (29%)	22 (17%)	10 (21%)	10 (22%)
3	47 (25%)	32 (24%)	12 (25)	11 (24%)
4	42 (23%)	36 (28%)	17 (35%)	7 (16%)
5	24 (13%)	22 (17%)	5 (11%)	8 (18%)
More than 5	19 (10%)	18 (14%)	4 (8%)	9 (20%)
Duration of Parkinson's disease [*n* (%)]*				
Less than 2 years	39 (21%)	15 (11%)	6 (12%)	5 (11%)
2–5 years	65 (35%)	36 (28%)	18 (37%)	20 (44%)
6–10 years	53 (28%)	42 (32%)	13 (27%)	12 (27%)
More than 10 years	27 (14%)	37 (28%)	11 (23%)	8 (18%)
Level of education [*n* (%)]*				
Elementary/Middle School/High School	53 (29%)	38 (29)	17 (35%)	10 (22%)
Professional Education, Vocational Training, or University	132 (71%)	93 (71%)	31 (65%)	35 (78%)
Digital literacy [*n* (%)]*				
Low	14 (8%)	16 (12%)	11 (23%)	5 (11%)
Medium	19 (10%)	27 (20%)	11 (23%)	8 (18%)
High	152 (82%)	89 (68%)	26 (54%)	32 (71%)
WTP (€) monthly for the use of the RMS service [mean (SD)]*	63 (96)	47 (94)	21 (27)	25 (25)

*Significant differences among classes at the 5% level.

^a^ Base case scenario – monitoring frequency of 8 h daily, monthly 10-min online questionnaires, no video recordings at home, and a clinical benefit of delaying symptom progression by one year.

^b^ Video monitoring at home scenario – monitoring frequency of 8 h daily, monthly 10-min online questionnaires, video recordings at home, and a clinical benefit of delaying symptom progression by one year.

^c^ 3-years delay in onset of advanced symptoms scenario – monitoring frequency of 8 h daily, monthly 10-min online questionnaires, no video recordings at home, and a clinical benefit of delaying symptom progression by three years.

Class 2, representing 32% of the sample and labeled “Follow Me,” displayed a strong preference for home video recordings, the most important attribute for this group (Table 2, Figure 2). This group primarily consisted of individuals with longer disease duration and more advanced symptoms (Table 3).

Class 3, identified as “Monitoring Skeptics,” accounted for 12% of the sample and showed a strong preference for opting out of RMS, as indicated by a large positive opt-out coefficient (Table 2). Although clinical benefits, such as delaying advanced symptoms, were the most influential and positively perceived attribute for this group, the burden of monitoring appeared to deter engagement (Table 2, Figure 2). Respondents in this class tended to be older, had lower education levels, and exhibited the lowest WTP for RMS (Table 3).

Class 4, termed “Privacy-Conscious,” represented 11% of the sample and exhibited significant aversion to home video recordings, as evidenced by the strong negative coefficient for this attribute, which emerged as the most important attribute for this group (Table 2, Figure 2). This class predominantly consisted of female respondents with higher education levels (Table 3).

No significant associations were found between class membership and factors such as living situation, prior experience with wearable technology, or Finnish or Italian nationality.

The probability of adopting RMS varied significantly across the four classes and was influenced by changes in key attributes (Table 3). In Class 1, adoption probability under the base case scenario (monitoring frequency of 8 h daily, monthly 10-min online questionnaires, no home video recordings, and a clinical benefit of delaying symptom progression by one year) was 81%. This probability increased substantially to 99% when the delay in the onset of advanced symptoms was extended to three years. In Class 2, the base case adoption probability was at 90%, rising to 94% with the inclusion of home video recordings. In contrast, Class 3 exhibited the lowest adoption probability, with only 13% under the base case scenario. This probability increased to 30% when the delay in symptom progression was extended to three years. For Class 4, the adoption probability under the base case scenario was the highest, at 95%. However, it dropped significantly to 49% when home video recordings were introduced, reflecting a strong aversion to this feature.

## Discussion

Our study explored PwP preferences for RMS using a DCE, a well-established and sophisticated quantitative method for eliciting preferences. A key finding was the considerable heterogeneity in RMS preferences among PwP, reflecting the complexity of decision-making in RMS adoption. While PwP generally demonstrated a great willingness to adopt RMS, preferences for specific attributes varied significantly across subgroups. This variation likely reflects differences in perceived benefits influenced by individual coping strategies, symptom severity, and personal perspectives.^37,38^

Consistent with previous studies,^39^ our results demonstrated that clinical benefits play a pivotal role in driving RMS adoption. Specifically, the delay in the onset of advanced symptoms emerged as the most important attribute for the “Benefit Seekers” subgroup, which constituted 45% of the sample. This subgroup primarily consisted of individuals in the earlier stages of PD or those experiencing milder symptoms, emphasizing the critical importance of demonstrable clinical outcomes in promoting RMS adoption within this population. Additionally, the limited interest in other RMS features observed among the “Benefit Seekers” aligns with prior qualitative research,^26^ which suggests that many PwP are willing to accept potential inconveniences, such as extended device usage or reduced privacy, in exchange for meaningful health improvements.

A second significant subgroup (“Follow Me”, 32%) exhibited a strong preference for home video recordings, which increased their adoption probability to 95% when included. This group, characterized by individuals with more advanced symptoms and longer disease durations valued the benefits of close monitoring. These findings align with prior evidence indicating that PwP experiencing greater disease burden prioritize features that support daily functioning and long-term autonomy, particularly when adequate privacy safeguards are implemented.^40^ The preference for video recordings may reflect a pragmatic recognition of Parkinson's disease as a progressive condition with no curative treatments, emphasizing the value of tools that enable monitoring and ongoing support to maintain independence.^41^

In contrast, the “Monitoring Skeptics” subgroup (12%) expressed a general reluctance toward RMS adoption. While clinical benefits were the most influential attribute for this group, the adoption probability remained low, with an increase from 13% to 30% when advanced symptoms delay was extended to 3 years. This group, characterized by older age, lower education levels, and the lowest WTP, reflects the challenges of engaging patients who may perceive RMS as burdensome or unnecessary. Previous research suggests that older adults often exhibit anxiety or feel overwhelmed by new technologies, which may explain their hesitation to engage with RMS.^42,43^ To address these barriers, tailored communication and educational strategies focusing on the usability, benefits, and support provided by RMS are essential for increasing acceptance in this segment.

Finally, the “Privacy-Conscious” (11%) demonstrated significant aversion to home video monitoring, likely due to privacy concerns and uncertainty regarding data security and its subsequent use, issues well-documented in the literature.^37,39,41^ Despite their strong negative response to video monitoring, this subgroup engaged meaningfully with other RMS attributes, particularly clinical benefits and monitoring frequency, suggesting a nuanced decision-making process. These findings highlight the importance of balancing the integration of advanced monitoring features with robust privacy safeguards to build trust and address concerns about data integrity.

The heterogeneity in preferences observed across subgroups has important implications for the design and implementation of RMS. First, clinical benefits should remain central to RMS development, as they emerged as the most influential factor positively driving adoption in over half of the study population. Second, features such as home video monitoring have the potential to increase adoption among PwP experiencing greater disease burden. However, this feature must be implemented with careful attention to privacy concerns to avoid deterring “Privacy-Conscious” users. Third, addressing the challenges faced by “Monitoring Skeptics,” particularly older adults, requires tailored interventions. These should include educational strategies, user-friendly interfaces, and targeted communication aimed at reducing anxiety and emphasizing the practical benefits of RMS.

While the study provides valuable insights, limitations should be acknowledged. First, the sample was limited to Finland and Italy, restricting the generalizability of the findings to other cultural or healthcare contexts. Second, recruiting participants through an online survey may have skewed the sample toward a more tech-savvy demographic, as reflected in the high proportion of well-educated respondents and low incidence of poor health literacy. Further research should aim to include more diverse populations to explore preferences across broader cultural, and digital literacy contexts. Third, although DCEs are a robust method for eliciting preferences, and evidence supports their ability to predict healthcare choices accurately,^44^ they do not explore the underlying motivations driving these preferences. To address this gap, further research incorporating qualitative methods is necessary to uncover the reasons behind the observed patterns, particularly when findings appear counterintuitive, and to validate the some of the explanations proposed in this study. Lastly, despite efforts to simplify the survey, some participants, particularly in Classes 1 and 2, appeared to focus on a single attribute. While this may reflect genuinely strong preferences, it could also indicate non-compensatory decision-making strategies driven by cognitive impairment or difficulty to understand and complete the survey.^45^ To address this, future studies could incorporate cognitive screening tools to ensure participants can fully understand and engage with the survey, thereby improving the precision and validity of preference estimates in PD contexts.^46^

## Conclusion

Our findings demonstrate that while PwP generally view RMS favorably, preferences for specific attributes vary significantly across subgroups. This variability underscores the limitations of a one-size-fits-all approach to RMS implementation in PD management. Instead, tailored system designs that accommodate the diverse preferences and expectations of different patient subgroups are essential to maximize user acceptance and optimize the effectiveness of RMS in supporting PD care.

## Supplemental Material

sj-docx-1-pkn-10.1177_1877718X251327752 - Supplemental material for Diverse preferences, different solutions: Exploring remote monitoring preferences in Parkinson's disease through a discrete choice experiment Supplemental material, sj-docx-1-pkn-10.1177_1877718X251327752 for Diverse preferences, different solutions: Exploring remote monitoring preferences in Parkinson's disease through a discrete choice experiment by Carlos Antonio Godoy Junior, Laura Mäkitie, Eleonora Fiorenzato, Maija Koivu, Joonas Niskala, Angelo Antonini, Lytske Jantien Bakker, Luis Pilli, Carin Uyl-de Groot, William Ken Redekop and Welmoed Kirsten van Deen in Journal of Parkinson's Disease

sj-docx-2-pkn-10.1177_1877718X251327752 - Supplemental material for Diverse preferences, different solutions: Exploring remote monitoring preferences in Parkinson's disease through a discrete choice experiment Supplemental material, sj-docx-2-pkn-10.1177_1877718X251327752 for Diverse preferences, different solutions: Exploring remote monitoring preferences in Parkinson's disease through a discrete choice experiment by Carlos Antonio Godoy Junior, Laura Mäkitie, Eleonora Fiorenzato, Maija Koivu, Joonas Niskala, Angelo Antonini, Lytske Jantien Bakker, Luis Pilli, Carin Uyl-de Groot, William Ken Redekop and Welmoed Kirsten van Deen in Journal of Parkinson's Disease

sj-docx-3-pkn-10.1177_1877718X251327752 - Supplemental material for Diverse preferences, different solutions: Exploring remote monitoring preferences in Parkinson's disease through a discrete choice experiment Supplemental material, sj-docx-3-pkn-10.1177_1877718X251327752 for Diverse preferences, different solutions: Exploring remote monitoring preferences in Parkinson's disease through a discrete choice experiment by Carlos Antonio Godoy Junior, Laura Mäkitie, Eleonora Fiorenzato, Maija Koivu, Joonas Niskala, Angelo Antonini, Lytske Jantien Bakker, Luis Pilli, Carin Uyl-de Groot, William Ken Redekop and Welmoed Kirsten van Deen in Journal of Parkinson's Disease

sj-docx-4-pkn-10.1177_1877718X251327752 - Supplemental material for Diverse preferences, different solutions: Exploring remote monitoring preferences in Parkinson's disease through a discrete choice experiment Supplemental material, sj-docx-4-pkn-10.1177_1877718X251327752 for Diverse preferences, different solutions: Exploring remote monitoring preferences in Parkinson's disease through a discrete choice experiment by Carlos Antonio Godoy Junior, Laura Mäkitie, Eleonora Fiorenzato, Maija Koivu, Joonas Niskala, Angelo Antonini, Lytske Jantien Bakker, Luis Pilli, Carin Uyl-de Groot, William Ken Redekop and Welmoed Kirsten van Deen in Journal of Parkinson's Disease

sj-docx-5-pkn-10.1177_1877718X251327752 - Supplemental material for Diverse preferences, different solutions: Exploring remote monitoring preferences in Parkinson's disease through a discrete choice experiment Supplemental material, sj-docx-5-pkn-10.1177_1877718X251327752 for Diverse preferences, different solutions: Exploring remote monitoring preferences in Parkinson's disease through a discrete choice experiment by Carlos Antonio Godoy Junior, Laura Mäkitie, Eleonora Fiorenzato, Maija Koivu, Joonas Niskala, Angelo Antonini, Lytske Jantien Bakker, Luis Pilli, Carin Uyl-de Groot, William Ken Redekop and Welmoed Kirsten van Deen in Journal of Parkinson's Disease
