# Supplementary material for: Diverse preferences, different solutions: Exploring remote monitoring preferences in Parkinson's disease through a discrete choice experiment
Source: J Parkinsons Dis. 2025 Mar 24:1877718X251327752. Online ahead of print. doi: 10.1177/1877718X251327752 (PMC13347457; doi:10.1177/1877718X251327752)
Supplement: Supplementary file 1 [file sj-docx-1-pkn-10.1177_1877718X251327752.docx]

**Supplementary file 1**

**Translated version of the whole survey**

Welcome to the survey

Login

Understanding Parkinson's disease patients' preferences for remote monitoring

The use of remote monitoring with smartwatches, smartphones and sensors at home can help patients with Parkinson's disease to have a better life. A large number of devices and apps to monitor Parkinson's symptoms at home are now available. However, research is needed to determine the characteristics of remote monitoring options that are acceptable to patients.

Next

Aim

Hereby we invite you to participate in our survey, which takes about 15-20 minutes. The goal of this survey is to understand your preferences for different remote monitoring options. This research is promoted by the University of Padova (Italy), Helsinki University Hospital (Finland) and Erasmus University Rotterdam (The Netherlands). The information you provide will be used to improve the diagnosis and treatment of Parkinson's disease. Please, answer all questions as accurately as possible.

Back

Next

0

%

100

%

Informed consent

Before you start with the survey, please read the information sheet and give your consent to participate.

Check all the boxes if you agree and would like to proceed with the survey.

My re~~s~~ponses will be collected anonymously and will only be published/shared in aggregated or anonymous form in scholarly publications and project reports.

My participation in this survey is completely voluntary.

I do not need to answer any questions which I don't want to answer, and I can quit at any time while filling in the survey. If I decide to cease participation, there will be no adverse consequences for me.

I will not receive payment for taking part of this study.

My confidential information or personal data will not be publicized in any way: no one will be able to trace this information back to me.

Back

Next

0% 100%

Parkinson's related questions

Stage

We would like to start the survey with few questions related to your current condition.

The best-known symptoms of Parkinson's disease involve movement-related symptoms, such as slow movements, tremors, and muscle stiffness. Symptoms can be categorized into mild, moderate, and advanced.

Mild symptoms: Symptoms are a bother, but they usually don’t interfere with your daily activities. Medication works well to keep them under control, and you usually don't need help with your daily activities.

Moderate symptoms: You begin to experience more symptoms and restrictions in some daily activities such as cycling or playing sports. You may find that the medication you take starts to decrease in efficiency between doses. You may need some help with your daily activities.

Advanced symptoms: Symptoms get worse. You experience problems with simple daily tasks, such as buttoning a shirt, because of your symptoms. The medication doesn’t work well anymore, and you start to experience fluctuations, where suddenly the symptoms are much more noticeable, and movement becomes more difficult. You often need help with your daily activities.

How would you classify your Parkinson's symptoms?

Back

Next

Parkinson's related questions

ParkinsonQ1

How long have you been diagnosed with Parkinson's disease?

Less then 2 years

Between 2-5 years

Between 6-10 years

More then 10 years

How many times per day do you take Levodopa (Levodopa-Benserazide, LevodopaCarbidopa-Entacapone, Madopar®, Nakon®, Sinemet®, Stalevo®, Pentiro®)?

2 or less times per day
3 times per day
4 times per day
5 times per day
6 or more times per day

Back

Next

0% 100%

Parkinson's related questions

ParkinsonQ3

Did you ever receive or are currently receiving treatment with deep brain stimulation?

Did you ever receive or are receiving treatment with levodopa pump?

Back

Next

0% 100%

DemographicQ1

Now, we would like to ask you a few general questions.

What is your age?

DemographicQ2

What is your gender?

Back

Next

DemographicQ3

What is your ethnic group?

Tick on the option that best describes your ethnic group or background.

Back

Next

0% 100%

DemographicQ4

What is the highest level of education you have completed?

Less than high school

High school

Vocational education, professional education or University

What is your current living situation?

I live in a household

I live alone

Back

Next

0% 100%

DemographicQ6

Which of this best describes the general area where you live?

City or town

Rural or remote area

Do you receive any form of professional care at home (e.g. home care aides, nursing, frequent	general practitioner visits at home)?

Back

Next

Survey information

SurveyInfo1

Shortly, we will begin with the questions to understand your preferences for remote monitoring.

Each remote monitoring option that we will show has four main characteristics. The first two characteristics are described below. Important: At this moment, we are only presenting the characteristics. You don't have to make any choices yet.

1. Monitoring intensity - The minimum number of hours per day you must wear a wearable device (e.g., a wristband) during one week each month. The alternatives are:
   - 8 hours a day for one week per month
   - 12 hours a day for one week per month
   - 24 hours a day for one week per month
2. Time filling questionnaires - The amount of time that you spend each month completing questionnaires about your symptoms in an online portal. The alternatives are:
   - 10 minutes per month
   - 20 minutes per month
   - 30 minutes per month

Back

Next

Now let's explore the remaining characteristics of each remote monitoring option. Please remember that this page is solely for informational purposes, and you do not need to make any decisions at this time.

3. Video recordings - The use of video recording systems to capture footage of your movements around your home. The alternatives are:

- Yes

- No

4. Delay in onset of advanced symptoms - The delay in the onset of advanced Parkinson's symptoms as a result of using remote monitoring. Remote monitoring allows close monitoring of symptoms, medication adherence, and other important factors. This information helps your doctor adjust your treatment plan and provide optimized care. By doing so, remote monitoring has the potential to slow down the progression of the disease and delay advanced symptoms. The alternatives are:

- 1 year delay
- 2 years delay
- 3 years delay

Back

Next

0% 100%

You are familiar with the four main characteristics for each remote monitoring option. Below, you'll see a list of those four characteristics.

Which characteristic matters most to you when picking a remote monitoring option?

Video recordings

Delay in onset of advanced symptoms

Time filling questionnaires

Monitoring intensity

Back

Next

0% 100%

Now that you are familiar with all the characteristics that make up the remote monitoring options, let's proceed with a final warm-up question to help you become more comfortable with the survey.

Imagine your doctor recommends you to use remote monitoring to track your Parkinson's symptoms.

Which of the options below would you select? (1 of 1)

Back

Next

0%	100%

SurveyInfo3

Great work! You're done with the practice questions. Now, let's dive into the main ones.

During the remainder of this questionnaire, we will show you twelve scenarios, where you will be asked to choose between two remote monitoring options with different characteristics. Please tell us which remote monitoring option you would choose. If you would not choose any of the remote monitoring options presented, please select the last option ("None").

Back

Next

0% 100%

# (1 of 12)

Back

Next

(2 of 12)

Back

Next

(3 of 12)

Back

Next

(4 of 12)

Back

Next

(5 of 12)

Back

Next

(6 of 12)

Back

Next

LiteracyQ1

You have completed the first half of the preferences questions. We would like to ask you some questions related to your use of digital technologies.

Do you use a wearable device (e.g., Fitbit, Apple Watch, Garmin, Polar, Samsung

Gear)

Yes

No

I don't know what a wearable device is.

Back

Next

0% 100%

LiteracyQ2

How often do you use a computer or smartphone?

Never

Occasionally

A few times per month

A few times per week

Everyday

How confident are you filling out online medical questionnaires by yourself?

Not at all

A little bit

Somewhat

Quite a bit

Extremaly

Click next to continue with the preference questions!

Back

Next

# (7 of 12)

Back

Next

(8 of 12)

Back

Next

(9 of 12)

Back

Next

(10 of 12)

Back

Next

(11 of 12)

Back

Next

(12 of 12)

Back

Next

WTP

You have completed all the preference questions. Thank you! We have just few more questions before we finish the survey.

From your choices, it seems you have a preferred remote monitoring system in mind. Now, let's imagine the hospital has a tight budget and can't afford to provide this technology to everyone without a cost. If they gave you an opportunity to rent this system (like a wristband, tablet, and camera) for six months, how much would you feel comfortable paying each month in Euros (€)?

€

Back

Next

0% 100%

QoL

These are our last questions.

Due to having Parkinson's disease, how often during the last month have you......

| Never | Occasionally Sometimes | Often | Always |
| --- | --- | --- | --- |

Back

Next

End of the survey

Final

Thank you for taking this survey, please submit your responses by clicking "EXIT" below.

EXIT

0% 100%
