# Supplementary material for: Diverse preferences, different solutions: Exploring remote monitoring preferences in Parkinson's disease through a discrete choice experiment
Source: J Parkinsons Dis. 2025 Mar 24:1877718X251327752. Online ahead of print. doi: 10.1177/1877718X251327752 (PMC13347457; doi:10.1177/1877718X251327752)
Supplement: Supplementary file 2 [file sj-docx-2-pkn-10.1177_1877718X251327752.docx]

**Supplementary file 2**

**Accounting for scale heterogeneity between the Finnish and Italian datasets – heteroskedastic model (**λ**)**

| Attributes | Levels | MNL | | |
| --- | --- | --- | --- | --- |
|  |  |  | | |
|  |  | **Estimate** | **SE** | **p value** |
| Monitoring frequency | 8h a day | *Ref.* | *..* | *..* |
|  | 12h a day | *-0.02* | *0.06* | *0.62* |
|  | 24h a day | *-0.14* | *0.05* | *0.04*** |
| Time filling online questionnaires | 10 minutes | *Ref.* | *..* | *..* |
|  | 20 minutes | *0.05* | *0.04* | *0.17* |
|  | 30 minutes | *-0.13* | *0.05* | *0.00**** |
| Video recordings at home | No | *Ref.* | *..* | *..* |
|  | Yes | *-0.03* | *0.04* | *0.60* |
| Delay in onset of advanced symptoms | 1 year delay | *Ref.* | *..* | *..* |
|  | 2 years delay | *0.65* | *0.05* | *0.00**** |
|  | 3 years delay | *1.28* | *0.07* | *0.00**** |
| Opt-out | | *-0.62* | *0.07* | *0.00**** |
| Lambda (λ) | | *0.05* | *0.05* | *0.67* |

**Significant at the 10% level; **Significant at the 5% level; ***Significant at the 1% level.*

The **lambda (λ) parameter** represents **scale heterogeneity** in a discrete choice experiment (DCE), capturing variability in the consistency of respondents' choices.

**Mathematical Representation**

- The utility for a choice alternative i is modeled as: Ui=λ(β′Xi)+ϵi, Where:
- λ: Scale parameter (adjusting the deterministic utility component β′Xi.
- β′Xi: The deterministic component of utility (attributes and preferences).
- ϵi: Random error term, scaled by λ.

Estimating λ allows the model to distinguish variations in decision consistency (scale effects).

**Application to This Study**

In our analysis, the differences in λ between the Finnish and Italian models were minimal and not statistically significant. This indicates that scale effects—variability in how consistently respondents process and evaluate choices—did not differ between the two populations.
