# Supplementary material for: Diverse preferences, different solutions: Exploring remote monitoring preferences in Parkinson's disease through a discrete choice experiment
Source: J Parkinsons Dis. 2025 Mar 24;15(3):619–29. doi: 10.1177/1877718X251327752 (PMC13347457; doi:10.1177/1877718X251327752)
Supplement: sj-docx-3-pkn-10.1177_1877718X251327752 - Supplemental material for Diverse preferences, different solutions: Exploring remote monitoring preferences in Parkinson's disease through a discrete choice experiment [file sj-docx-3-pkn-10.1177_1877718X251327752.docx]

**Supplementary file 3**

**Latent class model (LCA)**

**Calculating number of classes**

| **Table1.** Optimal class number determination within the LCA model guided by information criteria indicators. | | | | | |
| --- | --- | --- | --- | --- | --- |
| Classes | LL | Nparam | AIC | BIC | CAIC |
| 2 | -3614 | 17 | 7262 | 7331 | 7348 |
| 3 | -3190 | 26 | 6433 | 6537 | 6563 |
| 4 | -3023 | 35 | 6116 | 6257 | 6292 |
| 5 | -2933 | 44 | 5954 | 6131 | 6175 |
| 6 | -2872 | 53 | 5851 | 6064 | 6117 |

LL-Log Likelihood, Nparam-number of parameters, AIC-Akaike Information Criterion, BIC-Bayesian Information Criterion, CAIC-Consistent Akaike Information Criterion.

**Figure1.** Optimal class number determination within the LCA model guided by information criteria indicators.

AIC-Akaike Information Criterion, BIC-Bayesian Information Criterion, CAIC-Consistent Akaike Information Criterion.

| **Table2.** Optimal class number determination within the LCA model guided by class share. | | | | | | |
| --- | --- | --- | --- | --- | --- | --- |
| 2 Classes | Class1 | Class2 |  |  |  |  |
| Class Share | 54% | 46% | .. | .. | .. | .. |
| N | 222 | 189 | .. | .. | .. | .. |
|  |  |  |  |  |  |  |
| 3 Classes | Class1 | Class2 | Class3 |  |  |  |
| Class Share | 46% | 34% | 20% | .. | .. | .. |
| N | 189 | 140 | 82 | .. | .. | .. |
|  |  |  |  |  |  |  |
| 4 Classes | Class1 | Class2 | Class3 | Class4 |  |  |
| Class Share | 45% | 32% | 12% | 11% | .. | .. |
| N | 185 | 132 | 49 | 45 | .. | .. |
|  |  |  |  |  |  |  |
| 5 Classes | Class1 | Class2 | Class3 | Class4 | Class5 |  |
| Class Share | 38% | 29% | 13% | 11% | 9% | .. |
| N | 156 | 119 | 54 | 45 | 37 | .. |
|  |  |  |  |  |  |  |
| 6 Classes | Class1 | Class2 | Class3 | Class4 | Class5 | Class6 |
| Class Share | 33% | 20% | 19% | 10% | 9% | 9% |
| N | 136 | 82 | 78 | 41 | 37 | 37 |

N-number of respondents
