# Supplementary material for: Diverse preferences, different solutions: Exploring remote monitoring preferences in Parkinson's disease through a discrete choice experiment
Source: J Parkinsons Dis. 2025 Mar 24;15(3):619–29. doi: 10.1177/1877718X251327752 (PMC13347457; doi:10.1177/1877718X251327752)
Supplement: sj-docx-4-pkn-10.1177_1877718X251327752 - Supplemental material for Diverse preferences, different solutions: Exploring remote monitoring preferences in Parkinson's disease through a discrete choice experiment [file sj-docx-4-pkn-10.1177_1877718X251327752.docx]

**Supplementary file 4**

**Accounting for heterogeneity**

**Class membership allocation– latent class model (membership function)**

| **Table1.** class membership allocation determined by individual respondent characteristics. | | | | |
| --- | --- | --- | --- | --- |
| Variable | Class | Estimate | SE | p value |
| Age | Class1 | -0.04 | 0.00 | 0.00*** |
|  | Class2 | Ref. | .. | .. |
|  | Class3 | 0.035 | 0.00 | 0.00*** |
|  | Class4 | -0.03 | 0.00 | 0.00*** |
| Female | Class1 | 0.49 | 0.24 | 0.04** |
|  | Class2 | Ref. | .. | .. |
|  | Class3 | -0.07 | 0.37 | 0.83 |
|  | Class4 | 0.66 | 0.39 | 0.09* |
| Number of daily levodopa dosages | Class1 | -0.20 | 0.08 | 0.019** |
|  | Class2 | Ref. | .. | .. |
|  | Class3 | -0.10 | 0.15 | 0.52 |
|  | Class4 | 0.03 | 0.15 | 0.84 |
| Current symptoms | Class1 | -0.48 | 0.18 | 0.01** |
|  | Class2 | Ref. | .. | .. |
|  | Class3 | -0.31 | 0.36 | 0.38 |
|  | Class4 | -0.13 | 0.37 | 0.72 |
| Years with PD | Class1 | -0.50 | 0.14 | 0.00*** |
|  | Class2 | Ref. | .. | .. |
|  | Class3 | -0.24 | 0.26 | 0.32 |
|  | Class4 | -0.35 | 0.29 | 0.23 |
| Living alone | Class1 | -0.19 | 0.31 | 0.54 |
|  | Class2 | Ref. | .. | .. |
|  | Class3 | 0.03 | 0.73 | 0.96 |
|  | Class4 | 0.20 | 0.80 | 0.79 |
| Educational level | Class1 | 0.16 | 0.16 | 0.31 |
|  | Class2 | Ref. | .. | .. |
|  | Class3 | -0.09 | 0.37 | 0.79 |
|  | Class4 | 0.42 | 0.07 | 0.00*** |
| Quality of live (PDQ-8 score) | Class1 | -0.04 | 0.02 | 0.03** |
|  | Class2 | Ref. | .. | .. |
|  | Class3 | -0.04 | 0.03 | 0.17 |
|  | Class4 | 0.00 | 0.03 | 0.82 |
| Health literacy | Class1 | 0.44 | 0.11 | 0.00*** |
|  | Class2 | Ref. | .. | .. |
|  | Class3 | -0.29 | 0.23 | 0.21 |
|  | Class4 | 0.05 | 0.28 | 0.83 |
| Experience with wearables | Class1 | -0.30 | 0.22 | 0.19 |
|  | Class2 | Ref. | .. | .. |
|  | Class3 | 0.55 | 0.45 | 0.22 |
|  | Class4 | 0.27 | 0.64 | 0.67 |
| Willingness to pay (WTP) | Class1 | 0.01 | 0.00 | 0.01** |
|  | Class2 | Ref. | .. | .. |
|  | Class3 | -0.02 | 0.00 | 0.00*** |
|  | Class4 | -0.01 | 0.01 | 0.05* |
| Finnish nationality | Class1 | 0.15 | 0.25 | 0.55 |
|  | Class2 | Ref. | .. | .. |
|  | Class3 | -0.44 | 0.40 | 0.27 |
|  | Class4 | -0.29 | 0.43 | 0.51 |

*Significant at the 10% level; **Significant at the 5% level; ***Significant at the 1% level.
