# Supplementary material for: Diverse preferences, different solutions: Exploring remote monitoring preferences in Parkinson's disease through a discrete choice experiment
Source: J Parkinsons Dis. 2025 Mar 24;15(3):619–29. doi: 10.1177/1877718X251327752 (PMC13347457; doi:10.1177/1877718X251327752)
Supplement: sj-docx-5-pkn-10.1177_1877718X251327752 - Supplemental material for Diverse preferences, different solutions: Exploring remote monitoring preferences in Parkinson's disease through a discrete choice experiment [file sj-docx-5-pkn-10.1177_1877718X251327752.docx]

**Supplementary file 5**

**Willingness to adopt**

*Calculations: Probability of adopting RMS (Willingness to adopt)*

${{Prob scenario A=e}^{Vscenario A}}/{(e^{Vscenario A}+ e^{Vopt-out})}$

The probability of adoption for the **base case scenario** when presented as an alternative to opting out.

-Class 1:

V(base case scenario) = 0 🡪 Exp(0) = 1

V(opt-out) = -1.484 🡪 Exp(-1.484) = 0.2267

Probability = 1/(1+0.2267) = 0.8151 = 81%

-Class 2:

V(base case scenario) = 0 🡪 Exp(0) = 1

V(opt-out) = -2.26 🡪 Exp(-2.26) = 0.1043

Probability = 1/(1+0.1043) = 0.9055 = 90%

-Class 3:

V(base case scenario) = 0🡪 Exp(0) = 1

V(opt-out) = 1.84 🡪 Exp(1.84) = 6.2965

Probability = 1/(1+6.2965) = 0.1370 = 13%

-Class 4:

V(base case scenario) = 0 🡪 Exp(0) = 1

V(opt-out) = -3.07 🡪 Exp(-3.07) = 0.0464

Probability = 1/(1+0.0464) = 0.9556 = 95%

The probability of selecting **scenario with video monitoring included** when the alternative is opting out.

-Class 1:

V(with video monitoring) = -0.026 🡪 Exp(-0.026) = 0.9743

V(opt-out) = -1.484 🡪 Exp(-1.484) = 0.2267

Probability = 0.9743/(0.9743+0.2267) = 0.8112 = 81%

-Class 2:

V(with video monitoring) = 0.66 🡪 Exp(0.66) = 1.9347

V(opt-out) = -2.26 🡪 Exp(-2.26) = 0.1043

Probability = 1.9347/(1.9347+0.1043) = 0.9488 = 94%

-Class 3:

V(with video monitoring) = 0.24🡪 Exp(0.24) = 1.2712

V(opt-out) = 1.84 🡪 Exp(1.84) = 6.2965

Probability = 1.2712/(1.2712+6.2965) = 0.1679 = 16%

-Class 4:

V(with video monitoring) = -3.11 🡪 Exp(-3.11) = 0.0446

V(opt-out) = -3.07 🡪 Exp(-3.07) = 0.0464

Probability = 0.0446/(0.0446+0.0464) = 0.4901 = 49%

The probability of selecting **scenario with 3-years delay in onset of advanced symptoms included** when the alternative is opting out.

-Class 1:

V(3-years delay in onset of advanced symptoms) = 5.32 🡪 Exp(5.32) = 204.384

V(opt-out) = -1.484 🡪 Exp(-1.484) = 0.2267

Probability = 204.384/(204.384+0.2267) = 0.9988 = 99%

-Class 2:

V(3-years delay in onset of advanced symptoms) = -0.11 🡪 Exp(-0.11) = 0.8958

V(opt-out) = -2.26 🡪 Exp(-2.26) = 0.1043

Probability = 0.8958/(0.8958+0.1043) = 0.8957 = 89%

-Class 3:

V(3-years delay in onset of advanced symptoms) = 1🡪 Exp(1) = 2.7182

V(opt-out) = 1.84 🡪 Exp(1.84) = 6.2965

Probability = 2.7182/(2.7182+6.2965) = 0.3015 = 30%

-Class 4:

V(3-years delay in onset of advanced symptoms) = 0.9 🡪 Exp(0.9) = 2.4596

V(opt-out) = -3.07 🡪 Exp(-3.07) = 0.0464

Probability = 2.4596/(2.4596+0.0464) = 0.9814 = 98%
